# Antimicrobials use and resistance on integrated poultry-fish farming systems in the Ayeyarwady Delta of Myanmar

**DOI:** 10.1038/s41598-020-73076-2

**Published:** 2020-09-30

**Authors:** Justine S. Gibson, Honey Wai, Shwe Sin May Lwin Oo, Ei Moh Moh Hmwe, Soe Soe Wai, Lat Lat Htun, Hwee Ping Lim, Zin Min Latt, Joerg Henning

**Affiliations:** 1 School of Veterinary Science, The University of Queensland, Gatton, QLD Australia; 2 University of Veterinary Science, Yezin, Myanmar; 3 Livestock Breeding and Veterinary Department, Nay Pyi Taw, Myanmar

**Keywords:** Microbiology, Antimicrobials, Bacteria, Medical research, Epidemiology

## Abstract

Antimicrobials are used to support livestock health and productivity, but might pose a risk for the development of antimicrobial resistance; in particular, when multiple livestock species are raised together in production systems. On integrated chicken-fish farms, chickens are raised over fish ponds and poultry faeces is excreted into the ponds. We investigated antimicrobial usage and the antimicrobial susceptibility of *Escherichia coli* cultured from poultry faeces on 301 integrated farms in Ayeyarwady Delta of Myanmar. Antimicrobials were used by 92.4% of farmers for chickens, but they were not applied to fish. The most common antimicrobials used were Octamix (amoxicillin and colistin sulfate) on 28.4%, enrofloxacin on 21.0% and amoxicillin on 16% of farms. Overall, 83.1% (152/183) of the *E. coli* were resistant to at least one antimicrobial. The highest level of resistance was to amoxicillin (54.6%), tetracycline (39.9%), sulfamethoxazole/trimethoprim (35.5%) and enrofloxacin (34.4%). Multidrug resistance was identified in 42.4% of isolates. In general, we found similar levels of antimicrobial resistance in non-users of antimicrobials as in users of antimicrobials for more commonly applied antimicrobials. Overall, antimicrobial resistance was lower in chickens on these integrated farms in Myanmar, compared to poultry farms in other countries of South East and East Asia.

## Introduction

Antimicrobial agents such as beta-lactams, aminoglycosides, macrolides, and quinolones are used for growth promotion, prophylaxis and therapy in intensive poultry production systems in Southeast Asia^1^. This is of concern as the use of antimicrobials in food producing animals can select for antimicrobial resistant bacteria and resistance genes which can be transmitted from animals to humans via the food chain, direct contact and via the environment^2^. The World Health Organization (WHO) has recently highlighted the major public health risks posed by antimicrobial resistant bacteria and created a global action plan to combat antimicrobial resistance^3^.

The use of antimicrobial agents is of particular concern in systems where multiple livestock species are raised together and in systems, that are open to the surrounding environment. Such a production system is integrated chicken-fish farming, where chickens are raised over fishponds. Poultry faeces excreted into the ponds support photosynthetic organisms. Fish consume these organisms along with spilled chicken feed and faeces. The fish are often not fed any additional feed^4^ and water from the ponds is used to fertilise crops^5^. Thus integrated chicken-fish production provides an economical usage of land, labour and water^5^, but also raises a number of public health concerns because, antimicrobials, their residues, and antimicrobial-resistant pathogens or commensal organisms may accumulate in the environment promoting the development of antimicrobial-resistant bacteria^4^. Integrated chicken-fish farming plays a significant role in Myanmar’s economy, but the usage of antimicrobials and resistance to common bacteria is not known.

This study assessed the current practices of antimicrobial usage and antimicrobial susceptibility of *Escherichia coli* cultured from poultry faeces from integrated chicken-fish farms.

## Results

### Study population

A total of 304 integrated chicken-fish farmers were interviewed between February and July 2017 in the Maubin and Naungdon Townships of AD of Myanmar. Three farms did not have any poultry at the time of the interview and were excluded from the analysis. Of the 301 integrated chicken-fish farmers, 74.1% (N = 223) raised broilers, 23.9% (N = 72) raised layers, and 2.0% (N = 6) raised both broilers and layers. The four most common fish breeds on the farms were Rohu (79.1%, N = 238), Mrigal (59.1%, N = 178), Pacu (39.9, N = 120%), and Catla (35.2%, N = 106).

### Antimicrobial use in integrated poultry production

On broiler farms no antimicrobials were used on 10.8% (N = 24) farms, 33.2% (N = 74) used one antimicrobial and 56.0% (N = 125) used two or more antimicrobials. On layer farms no antimicrobials were used on 4.2% (N = 3) farms, 11.1% (N = 8) used one antimicrobial and 84.7% (N = 61) used two or more antimicrobials. Antimicrobials were used by all farms raising both broilers and layers (N = 6). Antimicrobials were more frequently used on layer compared to broiler farms (p < 0.001). All antimicrobials were administered in water.

The most common antimicrobials used were Octamix (amoxicillin and colistin sulfate) in 42.2% and 44.4% of broiler and layer flocks, respectively; enrofloxacin in 27.4% and 50.0%, and amoxicillin in 18.4% and 43.1%. Amoxicillin, enrofloxacin and tylosin were used more frequently (P < 0.05) in layer flocks compared to broilers flocks (Table 1), while some antimicrobials (neomycin, doxycycline, oxytetraclycine, lincomycin) were only used in broiler, but not in layer flocks.

**Table 1:** Cross-tabulation of types of antimicrobials used in broilers and layers on integrated chicken-fish farming systems in the Ayeyarwady Delta of Myanmar.

Antimicrobials	Overall (N = 295)		Broiler (N = 223)		Layer (N = 72)		P value
	N users	Percentage	N users	Percentage	N users	Percentage	
*Amoxicillin and colistin products*							
Amoxicillin	72	24.4	41	18.4	31	43.1	P < 0.001
Colistin	15	5.1	14	6.3	1	1.4	P = 0.128
Octamix (amoxicillin and colistin)	126	42.7	94	42.2	32	44.4	P = 0.785
*Fluoroquinolones (ciprofloxacin, enrofloxacin and ofloxacin)*							
Fluoroquinolone	7	2.4	4	1.8	3	4.2	P = 0.367
Ciprofloxacin	11	3.7	11	4.9	0	0.0	P = 0.071
Enrofloxacin	97	32.9	61	27.4	36	50.0	P = 0.001
Ofloxacin	24	8.1	21	9.4	3	4.2	P = 0.216
*Aminoglycosides*							
Neomycin	5	1.7	5	2.2	0	0.0	P = 0.340
Streptomycin	6	2.0	3	1.3	3	4.2	P = 0.158
*Tetracyclines*							
Doxycycline	11	3.7	11	4.9	0	0.0	P = 0.071
Oxytetracycline	1	0.3	1	0.4	0	0.0	P = 1.000
*Macrolide*							
Tylosin	56	19.0	28	12.6	28	38.9	P < 0.001
*Lincosamide*							
Lincomycin	7	2.4	7	3.1	0	0.0	P = 0.201
*Folic acid inhibitors*							
Sulfamethoxazole*/* trimethoprim	14	4.7	13	5.8	1	1.4	P = 0.200
Sulfadiazine	6	2.0	5	2.2	1	1.4	P = 1.000

Ocatmix, amoxicillin and tylosin were the antimicrobials most commonly used for treatments, while also being used prophylactically by other farmers. Streptomycin and sulfadiazine were predominately used for treatments only. Of all the farmers that provided octamix (42.7%, N = 126), about 43.7% (N = 55) administered it daily (Supplementary Table S1 online). In 99.3% of cases antimicrobials were obtained by integrated chicken-fish farmers from feed shops, and only on 0.7% of cases antimicrobials were supplied to farmers through veterinarians.

### Antimicrobial susceptibility testing

*Escherichia coli* was cultured from the faecal samples in 183 of 301 (60.8%) farms. Overall, 83.1% (152/183) of the *E. coli* were resistant to at least one antimicrobial (Table 2). The highest level of resistance was to ampicillin (54.6%), tetracycline (39.9%), sulfamethoxazole/trimethoprim (35.5%) and enrofloxacin (34.4%). Lower levels of resistance were identified to gentamicin (13.1%), cephalothin (10.2%), neomycin (6.6%), colistin (6.0%), amoxicillin clavulanic acid (3.8%) and cefoxitin (2.2%). No isolates were resistant to chloramphenicol. Multidrug resistance was identified in 65 isolates (42.4%). Many antimicrobial resistance patterns were detected (Supplementary Table S2 online).

**Table 2:** Antimicrobial susceptibility of 183 *E. coli* cultured from chicken faecal samples on integrated chicken-fish farming systems in the Ayeyarwady Delta of Myanmar.

Antimicrobial	Abbreviation	N farms (Percentage)		
		Susceptible	Intermediate	Resistant
Ampicillin/amoxicillin	AMP	58 (31.7)	25(13.7)	100 (54.6)
Amoxicillin/clavulanic acid	AMC	142 (77.6)	34 (18.6)	7 (3.8)
Cephalothin	KF	121 (66.1)	43 (23.5)	19 (10.2)
Cefoxitin	FOX	178 (97.3)	1 (0.5)	4 (2.2)
Ceftiofur	EFT	175 (95.6)	8 (4.4%)	0 (0)
Chloramphenicol	CHL	177 (96.7)	6 (3.3)	0 (0)
Colistin	Ct	172 (94.0)	^a^	11 (6.0)
Enrofloxacin	ENR	85 (46.4)	35 (19.1)	63 (34.4)
Gentamicin	GEN	154 (84.2)	5 (2.7)	24 (13.1)
Neomycin	N	177 (93.4)	^a^	12 (6.6)
Sulfamethoxazole/trimethoprim	SXT	112 (61.2)	6 (3.3)	65 (35.5)
Tetracycline	TET	107 (58.5)	3 (1.6)	73 (39.9)

^a^Colistin and neomycin only have susceptible and resistant clinical breakpoints.

### Relationship between antimicrobial use and antimicrobial resistance

**Figure 1:**
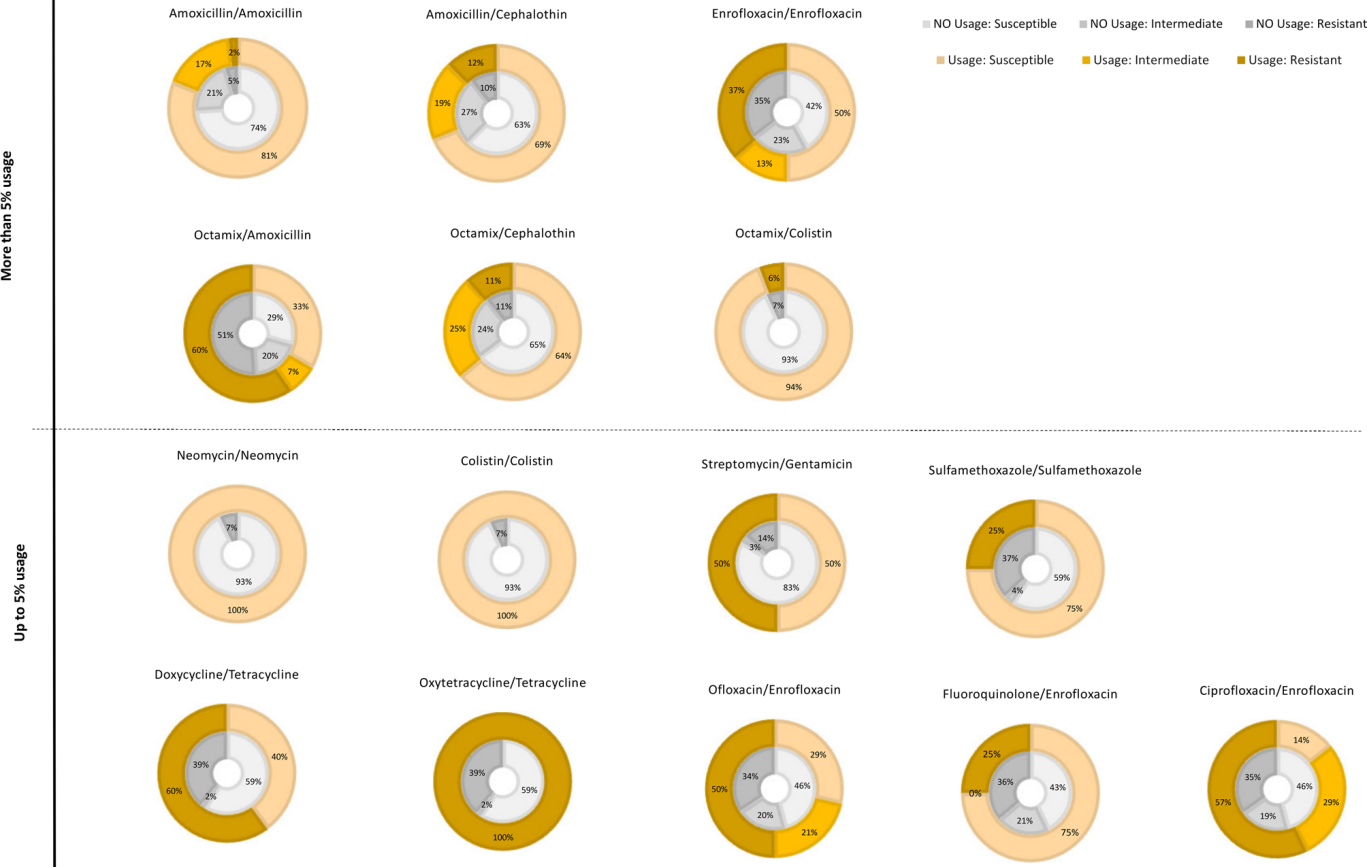
Resistance patterns (susceptible, intermediate, resistant) for antimicrobials that were used or not used on 172 integrated chicken-fish farms in Myanmar. Antimicrobials were separated in two groups, representing frequent application (> 5%) and infrequent application (< = 5%) across all integrated chicken-fish farms.

For 172 integrated chicken-fish farms the relationship between antimicrobial usage and antimicrobial resistance was explored. Focusing on antimicrobials with less than 95% susceptibility, we explored the relationship between usage and non-usage of antimicrobials in poultry on the integrated chicken-fish farms and the relevant resistance patterns for these antimicrobials. For more commonly applied antimicrobials (usage on more than 5% of farms), a similar resistance pattern was observed for users and non-users of this specific antimicrobial (Fig. 1), for example, amoxicillin resistance was similar for non-users and users of Octamix. For less commonly applied antimicrobials (usage in less than 5% of farms), more variability in antimicrobial resistance was observed between users and non-users.

**Figure 2:**
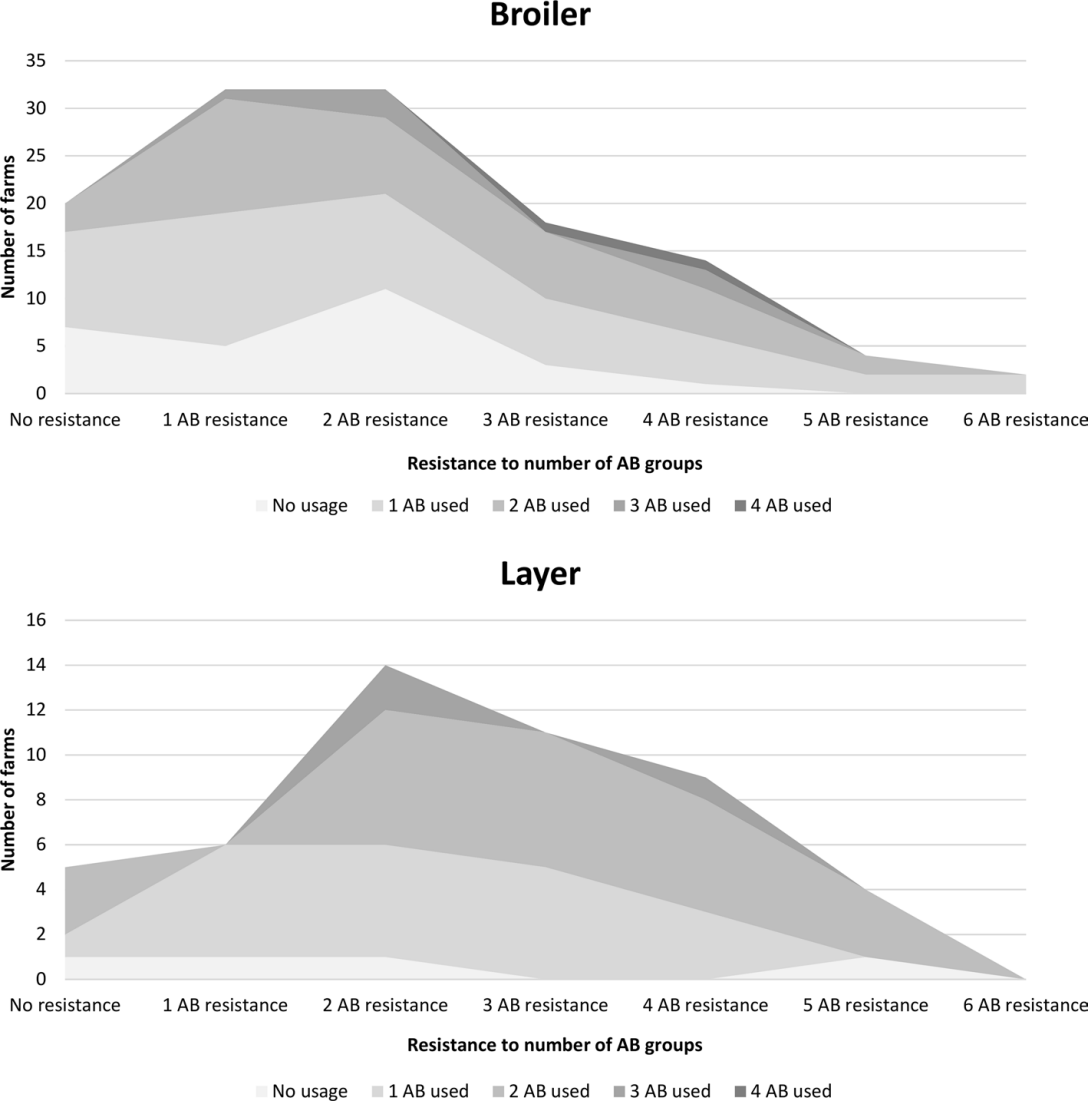
Relationship between the number of antimicrobials used on farms and the resistance to number of antimicrobial groups on these farms. Data is presented separately for layer and broilers raised on integrated chicken-fish farms in Myanmar.

About 16.4% of broiler and 10.2% of layer on the integrated chicken-fish farms showed no antimicrobial resistance, while resistance to one group or one antimicrobial category^6^ was 26.2% and 12.2% and for two or more antimicrobial categories was 57.4% and 77.6% for integrated broiler and layer farms, respectively (Fig. 2). Resistance to multiple antimicrobial categories was more prevalent on integrated layer compared to broiler farms (p = 0.048).

However, the correlation of the number of antimicrobials used on integrated chicken-fish farms did not show a linear relationship with the number of antimicrobial resistance groups found on these farms, thus a sequential larger number of antimicrobials used on integrated chicken-fish farms was not necessarily associated with a proportional increased resistance to the number of antimicrobial categories (Supplementary Fig. S1 online).

## Discussion

Antimicrobial resistance was found on integrated chicken-fish farms in Myanmar, but in general, we found lower levels of antimicrobial resistance in *E. coli* from healthy chickens compared with other countries in Southeast Asia^1,7–9^ and China^10,11^. In this study, the highest level of resistance was detected to older generation antimicrobials; ampicillin (54.6%), tetracycline (39.9%), and sulfamethoxazole/trimethoprim (35.5%).

Of most concern is the identification of resistance to the highest priority critically important antimicrobials, fluoroquinolones (e.g. enrofloxacin) (34.4%) and colistin (6%). These drugs are recognized as critically important for human therapy^12^ and the World Health Organisation recommends these drugs should not be used for treatment of food-producing animals”^13^. Fluoroquinolone resistance in this study was lower than that detected in faecal *E. coli* from chickens in Vietnam (59.6–70.2%)^8^ and Bangladesh (83–100%)^9^ and similar to a study from Indonesia (35.9%)^7^ and across studies from Southeast Asia > 20 to 50%^1^. Resistance to colistin in Southeast Asia in previous studies was reported as > 20 to 50%^1^.

Resistance to critically important third generation cephalosporins was low, cefoxitin (2.2%) similar to previous studies in Southeast Asia where levels < 10% were reported^1,8^. No third generation cephalosporins were reported as administered to chickens in this study. The macrolide, tylosin was also administered commonly to poultry in this study. Macrolides are generally administered to prevent or treat chronic respiratory disease (CRD) associated with *Mycoplasma gallisepticum*. Macrolides are also identified as highest priority critically important to human health^12^. Macrolides are generally not used to treat colibacillosis in poultry and no clinical breakpoints are available, therefore the faecal *E. coli* were not screened for susceptibility to macrolides. Future studies could involve culturing a gram-positive indicator organism e.g. *Enterococcus* spp. and screening to detect macrolide resistance.

A recent study on salmonella from poultry meat, from the Yangon Region Myanmar identified higher levels of resistance to sulfamethoxazole/trimethoprim (70.3%), tetracycline (54.3%), chloramphenicol (29.7%), amoxicillin clavulanic acid (17.4%) and multidrug resistance (52.2%) and lower levels of resistance to ampicillin (47.1%), ciprofloxacin (9.4%) and gentamicin (8%) than the current study^14^. Other studies in Myanmar have focused on MDR bacteria, extended spectrum beta-lactamase and carbapenemase producing Enterobacteriaceae from human clinical isolates^15–17^, or isolates cultured from the environmental^16^ or foods^18^.

In our study we did not test for carbapenems, however, we had no isolates resistant to ceftiofur, and only four isolates resistant to cefoxitin, three of which were MDR and MDR was identified in 42.4% of isolates.

Interestingly, we were able to show that for frequently used antimicrobials, similar antimicrobial resistance patterns were observed, even on those farms that did not use these antimicrobials. This suggests that a possible spread of antimicrobial resistance genes between farms for commonly used antimicrobials, including to farms that did not use specific antimicrobials. Our field data support and highlight the concerning relationship that antimicrobial resistance is a global public health concern as it did not stop at farm boundaries involving even farms that abstain of using specific antimicrobials.

However, our study also was not able to identify a clear linear correlation between antimicrobial use and resistance. A study investigating development of antimicrobial resistance in bacteria at integrated pig-fish farms in Vietnam described an increase in prevalence of naladixic acid and enrofloxacin resistance in faecal *E.coli* with enrofloxacin in feed, however the association between development of resistance and provision of feed containing enrofloxacin was not clear^19^. Tetracycline resistance was high prior to the study, and no association with provision of tetracycline in feed and increase in tetracycline resistance was identified^19^. However, the antimicrobial relationship between usage and resistance and spread of antimicrobial resistance genes is complex and is difficult to describe with field data, therefore modelling approaches and molecular studies are needed to illustrate the development of antimicrobial resistance over time.

We only used *E. coli* as an indicator pathogen for chicken faeces and did not sample the water in the ponds. Two types of indicator organisms for antimicrobial resistance in aquatic environments were used in another studies: *Acinetobacter* spp. and *Enterococcus* spp.^4^. In future studies *E. coli* and *Enterococcus* spp. could be used as indicator organisms in poultry and pigs while *Acinetobacter* spp. and *Enterococcus* spp. could be used for detection of resistance in the aquatic environment.

Overall, antimicrobial resistance was lower in broilers and layers on the studied integrated chicken-fish farms in Myanmar, compared to poultry farms in other countries of South East and East Asia. Although all famers used antimicrobials for poultry, none of the farmers used them for fish. Thus, chicken-fish farming in the study region of Myanmar seems to be a well-placed with respect to antimicrobial resistance, but adequate training of farmers is required to reduce the development and dissemination of antimicrobial resistance.

## Materials and methods

### Study design

A cross-sectional study was used to interview integrated chicken-fish farmers in the Ayeyarwady Delta (AD) of Myanmar. As no 'lists' or directories of integrated chicken-fish farmers existed, integrated chicken-fish farmers had to be located through participatory approaches. Thus, fishery officers from the Myanmar Livestock and Fisheries Department and veterinary officers from the Myanmar Livestock, Breeding and Veterinary Department ‘identified’ integrated chicken-fish farmers in selected townships by discussing with individual chicken or fish farmers where integrated chicken-fish farms were located. Integrated chicken-fish farmers were then visited and invited to participate in the interviews. Additional chicken-fish farmers were identified by snowball sampling, i.e. interviewed chicken-fish farmers provided information on other integrated chicken-fish farmers in the same township.

As the proportion of farms where *E. coli* is resistant to at least one antimicrobial was unknown, we assumed a prevalence of resistance 0.5, which would generate the largest sample size. A sample size of 302 was required to estimate a confidence interval up to 0.10 wide if the observed proportion is between 0.3 and 0.7 (Confidence level = 95%, Acceptable difference = 0.05, Assumed proportion = 0.5, Size of population = 1200). Sample size calculations were conducted in WinPepi version 11.65^20^.

### Questionnaire and data collection

A questionnaire was developed in English and digitized using the mobile data collection platform CommCare (Dimagi, Inc, https://www.commcarehq.org/home/). CommCare is an open source platform that allows the construction of specific mobile apps tailored for collection data. Digital tablets and smartphones were used to record data on the administration of antimicrobials (type, amount, type of administration, source), to collect spatial information on the farm location and to take photographs of labels of antimicrobials and supplements used by the farmers. Questionnaire data were collected by two veterinarians (who are co-authors of this paper) that were enrolled in master’s program at the University of Veterinary Science, Yezin, Myanmar at the time of the interviews.

Human Ethics Approval was obtained for this survey from the UQ Research and Innovation Ethical Review Committee (#2016001804). All research was performed in accordance with relevant guidelines/regulations of the Australian National Health and Medical Research Council Act. Informed consent was obtained from all interviewees (none of the interviewees were under 18, in which case, consent from a parent and/or legal guardian would have been required).

### Sample collection

Pooled chicken faecal samples were collected from the 301 integrated broiler and layer farms. Samples were collected from the slatted floor of the poultry sheds on each farm. Samples were transferred to a sterile container containing sterile saline (0.85% NaCl) and transported to the Department of Pharmacology and Parasitology, University of Veterinary Science (UVS), Yezin at 4 °C. The samples were stored at − 80 °C at UVS and sample processing commenced after the field data collection was completed in January 2018.

### Bacterial isolation

Samples were thawed at room temperature, vortexed and 100 µL spread onto MacConkey agar (MCA) plates (Oxoid, Basingstoke, United Kingdom). Plates were incubated aerobically at 37 °C for 24 h. One colony from each sample was selected on the basis of colony morphology and subcultured onto nutrient agar plates (Oxoid). Colonies were confirmed to be *E. coli* via API 20E testing (bioMerieux, Helios, Singapore). If the organism selected was not *E. coli*, another colony was selected. *E. coli* ATCC 25922 was used as a quality control organism.

### Antimicrobial susceptibility testing

Antimicrobial susceptibility testing was performed by disc diffusion as per Clinical and Laboratory Standards Institute guidelines (CLSI) for 12 antimicrobials of veterinary and/or human health importance: amoxicillin/clavulanic acid (30 μg); ampicillin (10 μg); cefoxitin (30 μg); ceftiofur (30 μg); cephalothin (30 μg); chloramphenicol (30 μg); colisitn (10 μg); enrofloxacin (5 μg); gentamicin (10 μg); neomycin (30 μg); sulfamethoxazole/trimethoprim (1.25/23.75 μg) and tetracycline (30 μg)^21^ between March 2017 and January 2018. All antimicrobial discs were obtained from Oxoid. The quality control organism used was *E. coli* ATCC 25922. Only enrofloxacin has a veterinary specific breakpoint for poultry^21^, therefore, human breakpoints were used for other antimicrobials^22^. For neomycin breakpoint information was obtained directly from the manufacturer (Zoetis, West Ryde, NSW, 2114, Australia). Isolates were considered multidrug resistant (MDR) if they acquired non-susceptibility to at least one agent in three or more antimicrobial categories^6^.

### Data analysis

Frequency and contingency tables and descriptive statistics were produced focusing on the usage of antimicrobials. The relationship between antimicrobial usage and resistance was visualized using a range of graphical displays and compared between integrated layer and broiler farms using the Fisher’s Exact test. Data analysis was conducted using Stata 14.0 (StataCorp, 4905 Lakeway Drive, College Station, Texas 77845 USA).

### Ethical statement

Institutional approval for conducting interviews was obtained from The University of Queensland, Human Research Ethics Committee (No. 2016001804).

## Supplementary information

Supplementary Tables S1 and S2.

Supplementary Figure S1.

## Data Availability

The dataset analysed in this study is available from the https://doi.org/ 10.6084/m9.figshare.11971995.
