# Supplementary material for: Antimicrobials use and resistance on integrated poultry-fish farming systems in the Ayeyarwady Delta of Myanmar
Source: Sci Rep. 2020 Sep 30;10:16149. doi: 10.1038/s41598-020-73076-2 (PMC7528085; doi:10.1038/s41598-020-73076-2)
Supplement: Supplementary file 1 — Supplementary Tables S1 and S2. [file 41598_2020_73076_MOESM1_ESM.pdf]

Antimicrobials use and resistance on integrated poultry-fish farming systems in the Ayeyarwady Delta of Myanmar

Justine S. Gibson[1*], Honey Wai[2], Shwe Sin May Lwin Oo[2], Ei Moh Moh Hmwe[2], Soe Soe Wai[2], Lat Lat Htun[2], Hwee Ping Lim[1], Zin Min Latt[3], Joerg Henning[1]

[1] School of Veterinary Science, The University of Queensland, Gatton, QLD, Australia

[2] University of Veterinary Science, Yezin, Myanmar

[3] Livestock Breeding and Veterinary Department, Nay Pyi Taw, Myanmar

Supplementary Table S1. Cross-tabulation of types of antimicrobials and medications used for poultry and frequency of application in integrated chicken-fish farms in the Ayeyarwady Delta of Myanmar.

Antimicrobials	Type of application						Total users
	Prophylactic - once daily		Use on "special" days		Therapeutic		
	N users	Percentage	N users	Percentage	N users	Percentage	
Amoxicillin and colistin products							
Amoxicillin	24	33.3	25	34.7	23	32.0	72
Colistin	7	46.7	3	20.0	5	33.3	15
Octamix (amoxicillin and colistin)	55	43.7	47	37.3	24	19.0	126
Fluoroquinolones (ciprofloxacin, enrofloxacin and ofloxacin)							
Fluoroquinolone	2	28.6	5	71.4	0	0.0	7
Ciprofloxacin	4	36.4	1	9.1	6	54.5	11
Enrofloxacin	29	29.9	56	57.7	12	12.4	97
Ofloxacin	12	50.0	3	12.5	9	37.5	24
Aminoglycosides							
Neomycin	3	60.0	2	40.0	0	0.0	5
Streptomycin	1	16.7	0	0.0	5	83.3	6
Tetracyclines							
Doxycycline	3	27.3	5	45.4	3	27.3	11
Oxytetracycline	0	0.0	1	100.0	0	0.0	1
Macrolide							
Tylosin	14	25.0	28	50.0	14	25.0	56
Lincosamide							
Lincomycin	2	28.6	2	28.6	3	42.8	7
Folic acid inhibitors							
Sulfamethoxazole/ trimethoprim	5	35.7	2	14.3	7	50.0	14
Sulfadiazine	1	16.7	1	16.7	4	66.6	6

Supplementary Table S2: Resistant phenotype of 183 ***E. coli*** cultured from chicken faecal samples on integrated chicken-fish farming systems in the Ayeyarwady Delta of Myanmar.

Antimicrobial categories	Multidrug resistant pattern	Number of isolates
**Resistance to six antimicrobial categories (N = 2)**		
Penicillins, tetracycline, folate pathway inhibitors, fluoroquinolone, aminoglycoside, polymyxins	AMP, TET, SXT, ENR, GEN,C	1
Penicllins, penicillins & β-lactam inhibitors, cephamycins, folate pathway inhibitors, fluoroquinolone, polymyxins	AMC, AMP, FOX, SXT, ENR, C	1
**Resistance to five antimicrobial categories (N = 8)**		
Penicllins, tetracycline, folate pathway inhibitors, fluoroquinolone, aminoglycoside	AMP, TET, SXT, ENR, GEN	**3**
	AMP, TET, SXT, ENR, N	1
	AMP, TET, SXT, ENR, GEN, N	1
Penicllins, penicillins & β-lactam inhibitors, folate pathway inhibitors, fluoroquinolone, aminoglycoside	AMP, AMC, SXT ENR, GEN	1
Penicillin, non-extended spectrum cephalosporin, tetracycline, folate pathway inhibitors, fluoroquinolone	AMP, KF, TET, SXT, ENR	2
**Resistance to four antimicrobial categories (N = 24)**		
Penicillins, tetracycline, folate pathway inhibitors, fluoroquinolone	AMP, TET, SXT, ENR	13
Penicillins, folate pathway inhibitors, fluoroquinolone, aminoglycoside	AMP, SXT, ENR, GEN	4
	AMP, SXT, ENR, N	1
Penicillins, aminoglycoside, tetracycline, folate pathway inhibitors	AMP,GEN,TET,SXT	1
Folate pathway inhibitors, fluoroquinolone, aminoglycoside, tetracycline	SXT, ENR, GEN, TET	2
Penicllins, penicillins & β-lactam inhibitors, tetracycline, folate pathway inhibitors	AMP, AMC, TET, SXT	1
Penicillins, non-extended spectrum cephalosporin, tetracycline, folate pathway inhibitors	AMP, KF, TET, SXT	1
Penicillin, non-extended spectrum cephalosporin, cephamycin, aminoglycoside	AMP, KF, FOX, GEN	1
**Resistance to three antimicrobial categories (N = 31)**		
Penicillin, tetracycline, folate pathway inhibitors	AMP, TET, SXT	7
Penicillin, tetracycline, fluoroquinolone	AMP, TET, ENR	4

Penicillins & β-lactam inhibitors, tetracycline, fluoroquinolone	AMC, TET, ENR	1
Penicillin, fluoroquinolone, folate pathway inhibitors	AMP, ENR, SXT	3
Pencillin, fluoroquinolone, aminoglycoside	AMP, ENR, GEN	1
	AMP, ENR, N	1
Folate pathway inhibitors, fluoroquinolone, aminoglycoside	SXT, ENR, GEN	2
	SXT, ENR, N	1
folate pathway inhibitors, fluoroquinolone, tetracycline	SXT, TET, ENR	1
Aminoglycoside, fluoroquinolone, tetracycline	GEN, ENR, TET	1
Polymyxin, folate pathway inhibitors, aminoglycoside	C, SXT,N	1
Penicllins, penicillins & β-lactam inhibitors, tetracycline	AMP, AMC, TET	1
Penicillins, non-extended spectrum cephalosporin, tetracycline	AMP, KF, TET	5
Non-extended spectrum cephalosporin, penicillins & β-lactam inhibitors, cephamycin	KF, AMC, FOX	1
Penicillins, non-extended spectrum cephalosporin, penicillins & β-lactam inhibitors	AMP, KF , AMC	1
**Resistance to two antimicrobial categories (N = 49)**		
Penicillin, tetracycline	AMP, TET	19
Penicillin, folate pathway inhibitors	AMP, SXT	4
Penicillin, aminoglycoside	AMP, N	1
	AMP, GEN	1
Non-extended spectrum cephalosporin, aminoglycoside	KF, GEN	1
Penicillin, fluoroquinolone	AMP, ENR	1
Non-extended spectrum cephalosporin, fluoroquinolone	KF, ENR	1
Penicillin, polymyxin	AMP, C	1
Non-extended spectrum cephalosporin, polymyxin	KF, C	1
Folate pathway inhibitor, fluoroquinolone	SXT, ENR	7
Folate pathway inhibitor, tetracycline	SXT, TET	2
Aminoglycoside, fluoroquinolone	N, ENR	1
	GEN, ENR	1
Polymyxin, Folate pathway inhibitor	C, SXT	2
Polymyxin, fluoroquinolone	C, ENR	1
Polymyxin, tetracycline	C, TET	1

Penicillin, non-extended spectrum cephalosporin	AMP, KF	3
Non-extended spectrum cephalosporin, cephamycin	KF, FOX	1
**Resistance to one antimicrobial categories (N = 38)**		
Penicillins	AMP	14
Non extended spectrum cephalosporin	KF	1
Fluoroquinolone	ENR	6
Tetracycline	TET	5
Aminoglycosides	N	4
	GEN	3
Polymyxin	C	3
Folate pathway inhibitor	SXT	2

AMP, ampicillin/amoxicillin; EFT, ceftiofur; KF, cephalothin; AMC, amoxicillin clavulanic acid; FOX, cefoxitin N, neomycin; GEN, gentamycin; TET, tetracycline; ENR, enrofloxacin; SXT, trimethoprim/sulfamethoxazole; C, Colistin

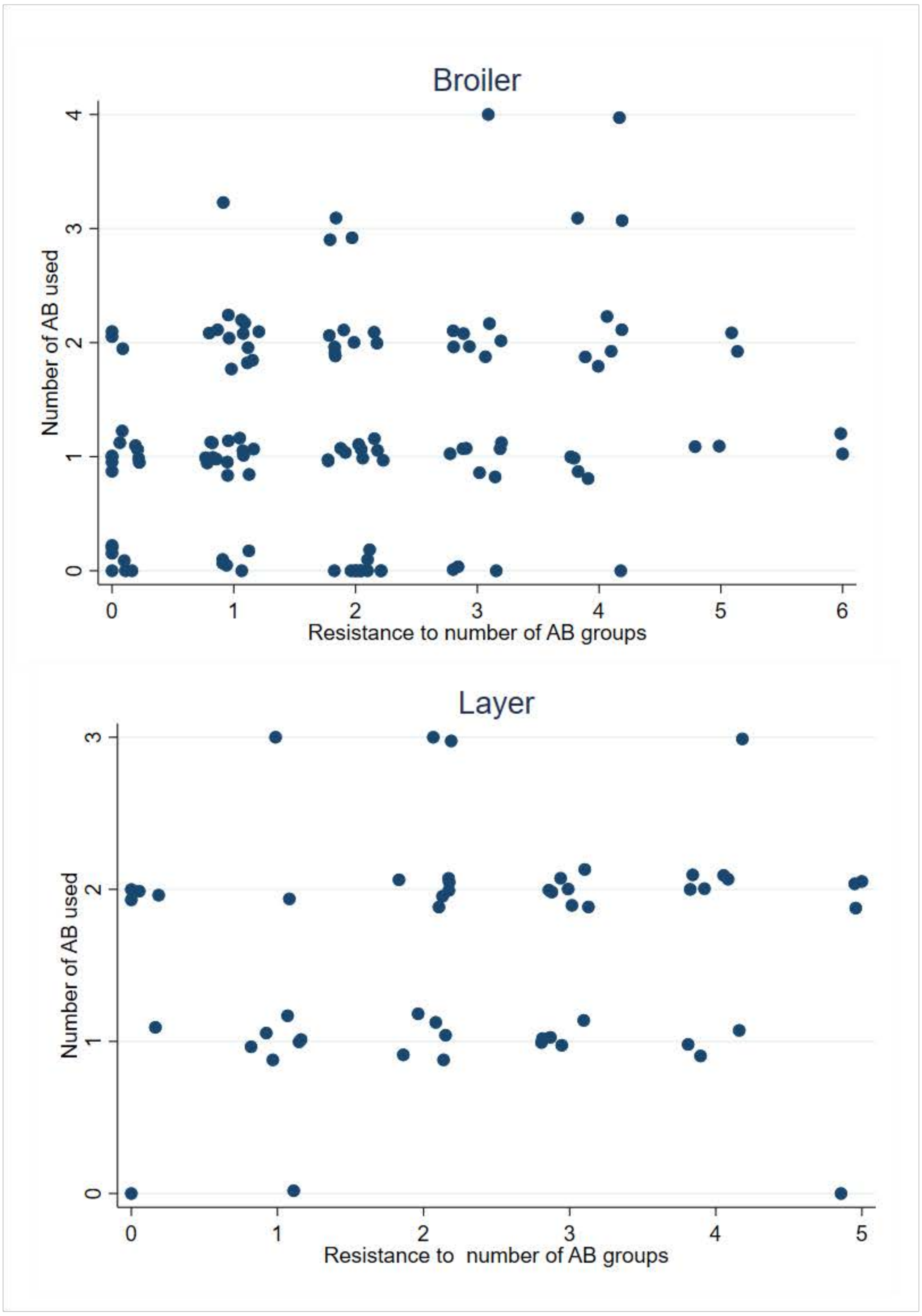

Supplementary Fig. S1
Scatterplots of the number or antimicrobials used on farms and the resistance to the number of antimicrobial groups on these farms. Data is presented separately for layer and broilers raised on integrated chicken-fish farms in Myanmar.
